# *FORC*^A^, a promoter element that responds to crosstalk between defense and light signaling

**DOI:** 10.1186/1471-2229-9-2

**Published:** 2009-01-07

**Authors:** Alexandre Evrard, Theogene Ndatimana, Thomas Eulgem

**Affiliations:** 1 Center for Plant Cell Biology, Institute for Integrative Genome Biology, Department of Botany and Plant Sciences, University of California, Riverside, CA 92521, USA; 2 INRA/CNRS-URGV, 2 Rue Gaston Crémieux, CP5708 91057, Evry, France

## Abstract

**Background:**

Recognition of pathogenic microorganisms triggers in plants comprehensive transcriptional reprogramming. In order to identify transcriptome-level control elements required for plant immune responses we are examining several sets of genes found by microarray experiments to be co-activated in *Arabidopsis thaliana *(Arabidopsis) seedlings infected with the oomycete *Hyaloperonospora parasitica*. Promoter motifs conserved in clusters of co-expressed genes may be involved in mediating coordinated gene activity patterns. Although numerous studies identified such conserved promoter motifs in co-expressed gene sets, reports confirming their function as regulatory elements are rare.

**Results:**

*FORC*^A ^is a hexameric promoter motif that is conserved in clusters of Arabidopsis genes co-expressed in response to fungal or oomycete pathogens as well as defined light treatments. *FORC*^A ^is generally more frequently present in Arabidopsis promoter regions than statistically expected. It constitutively interacts in a DNA-sequence specific manner with nuclear Arabidopsis proteins. These interactions are suppressed by defense-related stimuli and enhanced by prolonged exposure to constant light. Furthermore *FORC*^A ^mediates constitutive reporter gene expression in transiently transformed *Nicotiana benthamiana *leaves as well as in stably transformed Arabidopsis plants. Its responsiveness to defense-stimuli is modulated by the duration of light exposure. In plants grown under normal light conditions or constant darkness defense-related stimuli result in suppression of *FORC*^A^-mediated reporter gene expression, while in plants grown under constant light exposure, defense-induction results in enhanced *FORC*^A^-mediated expression. In addition, we found plants subjected to constant light exposure to exhibit reduced susceptibility to virulent *H. parasitica*.

**Conclusion:**

We propose that *FORC*^A ^is a regulatory cis-element that is present in a wide variety of Arabidopsis promoters. It integrates light- and defense-related signals and participates in adjusting the transcriptome to changes in environmental conditions.

## Background

Molecular recognition of pathogenic microorganisms triggers in plants comprehensive transcriptional reprogramming. A network of defense-regulators transduces information about the attacking microbe into appropriate transcriptional responses. Two functionally distinct classes of pathogen molecules are known to elicit plant immune responses [1-3]. Receptor-mediated recognition of pathogen/microbe associated molecular patterns (PAMPs/MAMPs) activates PAMP-triggered immunity (PTI), while recognition of pathogen effectors by plant disease resistance (R) proteins leads to effector-triggered immunity (ETI). ETI is a strong immune response that results in incompatible plant pathogen interactions where the plant is resistant and the pathogen is avirulent. Effectors are pathogen-derived molecules (proteins or small organic molecules) that are secreted into host tissues [4]. Some effectors have been shown to suppress PTI resulting in a weakened immune response called basal defense which is usually insufficient to halt growth and spread of the respective microbe. The typical outcome in this case is a compatible interaction, where the plant is susceptible and the pathogen is virulent.

A key player in the regulation of ETI as well as basal defense is the hormone salicylic acid (SA), which accumulates in infected tissues of several plant species and triggers downstream defense responses [5]. Several other small molecules and multiple proteins involved in defense regulation have been identified by genetic and biochemical studies [6]. In particular the immune system of *Arabidopsis thaliana *(Arabidopsis) has been extensively examined over the past two to three decades. While our knowledge about the Arabidopsis immune system is far from complete, it has become obvious in recent years, that its individual components are functionally interconnected in a complex manner and constitute a regulatory network rather than simple linear pathways. Crosstalk between distinct branches of this network has been found to be involved in fine-tuning defense outputs and maximizing the effectiveness of immune responses [7]. For example, sophisticated mechanisms mediating crosstalk between SA and jasmonic acid (JA)-dependent signaling processes can result in synergistic or antagonistic effects depending on the levels of each of these hormones [8-10].

In addition, there are extensive interactions of defense signaling with regulatory processes that are not primarily involved in plant immune responses. For example, crosstalk between defense mechanisms dependent on the defense hormones SA and JA on the one hand and each of the classical growth-controlling phytohormones, auxin, gibberellin, ABA, cytokinin or brassinosteroids on the other hand have been reported [11]. Details of these interactions are not well understood yet, but it seems that some of them benefit the host by enhancing immune responses, while others are beneficial for the pathogen by suppressing defense reactions. In addition, several labs reported interferences between light and defense signaling. Depending on the type of light signaling pathway involved, these interactions were found to be synergistic or antagonistic. For example, phytochrome-mediated light signaling can positively affect SA-dependent defense responses [12,13], while negative interferences were observed between defense and UV-light signaling [14]. Generally, these interactions seem to control resource allocation in the plant ensuring the maintenance of homeostasis and the adjustment of the host's metabolism to a state optimal to cope with the respective environmental challenges.

We are using interactions between the pathogenic oomycete *Hyaloperonospora parasitica *(*Hp*) and Arabidopsis to examine regulatory mechanisms operating at the interface between defense signaling and the regulation of the defense transcriptome [15-17]. Several transcriptional regulators have been implicated in these processes, such as NPR1, a nuclear transported transcriptional cofactor that acts downstream from SA and interacts with TGA-bZIP transcription factors [18]. Additional transcription factors, including WRKYs and ERFs, participate in the regulation of the plant defense transcriptome and disease resistance [19]. The DNA binding site preferences of these transcription factors have been well characterized. Typically TGA-bZIPs bind to TGA boxes (TGACG), WRKYs to W boxes (TTGACC/T) and ERFs to GCC boxes (AGCCGCC) [19]. These promoter sites have been shown in numerous studies to act as pathogen-responsive *cis*-elements mediating expression of individual defense-associated genes [19].

An enormous amount of global gene expression data from large-scale transcript profiling projects has accumulated during the past decade. These data allowed for the identification of gene clusters that are co-expressed under certain biological conditions and, hence, are likely to be subject to co-regulation by common mechanisms. Several studies revealed conserved sequence motifs in promoters of such co-regulated genes which may act as *cis*-elements mediating their coordinated activity [20,21]. In addition to already known *cis*-elements, some novel promoter motifs were found to be statistically enriched in co-expressed genes [15,22,23]. While in some cases, their function has been experimentally proven [22,23], reports confirming that such conserved promoter motifs are binding sites of nuclear proteins that can affect gene expression are still rare.

Here we report on the identification and functional characterization of a motif conserved in promoters of the *FORC *cluster, a set of Arabidopsis genes co-expressed in response to recognition of *Hp *as well as other oomycete and fungal pathogens. We found this motif, which we termed *FORC*^A^, to exhibit DNA sequence specific and differential interactions with nuclear Arabidopsis proteins. Furthermore, electrophoretic mobility shift assays as well as reporter gene assays showed that *FORC*^A ^integrates stimuli related to defense- and light signaling. *FORC*^A ^acts as a light-responsive enhancer, the activity of which is modulated by pathogen-perception. Under normal light conditions as well as constant darkness, *FORC*^A ^activity is reduced by recognition of *Hp *as well as application of SA, while under constant light conditions its activity is enhanced by defense induction. Consistent with the observed functional connection between light and defense signaling, we observed that plants kept under constant light exposure exhibited reduced susceptibility to a virulent *Hp *isolate.

## Results

### A cluster of Arabidopsis genes co-expressed during interactions with oomycete and fungal pathogens is enriched for the *FORC*^A ^promoter motif

Using Affymetrix microarrays we previously identified clusters of genes that are co-expressed during Arabidopsis immune responses [15]. Two of these clusters, cluster I and the *LURP *cluster (designated as cluster II in [15]), were defined by distinct patterns of coordinated transcriptional up-regulation during incompatible and compatible interactions with the *Hp *isolates *Hp*Emoy2, *Hp*Hiks1 and *Hp*Emco5. While *LURP *transcripts exhibited a kinetic pattern of late and sustained up-regulation accumulating strongly between 12 and 48 hpi [16,24], cluster I transcript levels tended to increase earlier and predominantly accumulated within the first 12 h after infection [15]. Genetic disruption of SA-signaling and R protein-mediated *Hp *recognition strongly attenuated or delayed the response of *LURP *genes, but had only a minor effect on cluster I responses. Inspecting publicly accessible microarray data (Botany Array Resource) [25] we found that cluster I genes also exhibit a pronounced pattern of co-activation during interactions of Arabidopsis with the fungal pathogen *Bortytis cinerea *as well as a second oomycete, *Phythophthora infestans *(see Additional File 1). Therefore we renamed cluster I to *FORC *(*Fungal and Oomycete Pathogen Response Cluster*). Although *LURP *genes are also coordinately activated in response to the oomycete *Phytohthora infestans*, this set does not show an uniform response to fungal pathogens (not shown).

Tightly co-expressed subsets of both the *FORC *and *LURP *genes were found to be enriched for distinct promoter motifs, such as potential binding sites of WRKY transcription factors [15]. This suggested that members of the *FORC *cluster on the one hand and the *LURP *cluster on the other hand are controlled by common regulatory mechanisms. The fact that different motifs are conserved in the promoters of each of these two gene sets suggested that the *FORC *genes are controlled by mechanisms distinct from those controlling the *LURP *genes. Applying AlignACE [26] to the 1000 bp-upstream sequences of a strictly co-expressed subset of *FORC *genes (Pearson correlation of 0.95 to the average pattern of the *FORC *set; [15]) we found the hexameric motif T/ATGGGC to be significantly enriched compared to its statistically expected frequency (*p *= 3.0 E-4; see under "Methods"). We termed this motif *FORC*^A^.

### *FORC*^A ^specifically interacts with a nuclear DNA binding activity that is down-regulated by *Hp *and SA

**Figure 1:**
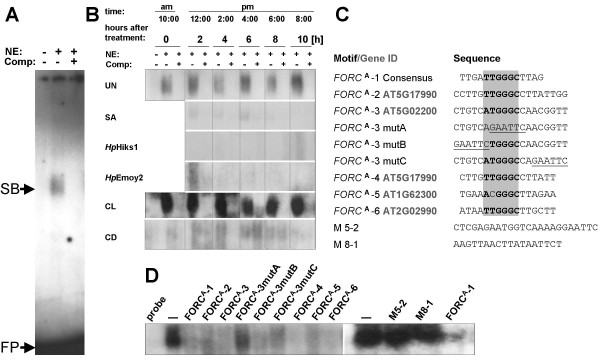
***In vitro*-interactions of *FORC*^A ^with nuclear Arabidopsis proteins**. A, B: Interactions of *FORC*^A ^with nuclear protein extracts from Arabidopsis Col-0 seedlings grown under normal light conditions and left untreated (UN) or treated with salicylic acid (SA), infected with the avirulent *Hp *isolates Hiks1 and Emoy2 or exposed for three days to continuous light (CL) or continuous darkness (CD). Panel (A) shows the whole gel for the 0 h untreated samples; panel (B) shows gel sections with the *FORC*^A^-specific band shift for all tested conditions. NE: nuclear extract, Comp: unlabeled competitor probe, SB: *FORC*^A^-specific band shift, FP: free probe.C: *FORC*^A ^derived oligonucleotides used in EMSAs. The gene IDs specify the promoters from which each motif is derived. Positions representing the defining T/ATGGGC core sequences of the shown wild type *FORC*^A ^permutations are highlighted by a grey box. Sequences mutated in the tested *FORC*^A^-3 permutations are underlined. D: EMSA competition analysis of *FORC*^A^-1 interactions using Arabidopsis nuclear protein extracts from Arabidopsis Col-0 seedlings exposed to CL.

T/ATGGGC-containing promoter motifs have not been described as pathogen-response elements. Therefore we tested by electrophoretic mobility shift assays (EMSAs), if *FORC*^A ^exhibits *Hp*-dependent differential interactions with nuclear Arabidopsis proteins (Figure 1). Using the radioactively labeled *FORC*^A^-1 probe consisting of a single copy of the *FORC*^A ^consensus sequence with the TTGGGC core and 8 bp of arbitrary flanking sequence we detected a constitutive DNA-binding activity producing a single band shift that is out-competed by unlabeled *FORC*^A^-1 probe (Figures 1A–C). Surprisingly, this *FORC*^A ^binding activity was down-regulated in response to infections with the avirulent *Hp *isolates *Hp*Hiks1 or *Hp*Emoy2 as well as exogenously applied SA. Furthermore, we found *FORC*^A^/nuclear protein interactions to be light dependent. Nuclear extracts from plants kept for three days under constant light produced substantially more intense *FORC*^A^-shifts than those from plants kept for the same time in constant darkness (CL & CD in Figure 1B). Besides unlabeled *FORC*^A^-1 probe, unlabeled competitor probes containing wild-type *FORC*^A ^permutations present in several *FORC *promoters (*FORC*^A^2–6) clearly reduced the intensity of *FORC*^A^-1 shifts (Figures 1C &1D). Mutations in the sequences flanking the invariant TGGGC core of *FORC*^A^-3 did not result in reduced competition. However, the *FORC*^A^-3 mutA competitor probe, that lacks the TGGGC core, as well as competitor probes with sequences unrelated to *FORC*^A ^(M5-2, M8-1) did not efficiently reduce the intensity of *FORC*^A^-1 band shifts. These results showed that the observed *FORC*^A^-binding activity is DNA-sequence specific and that only the TGGGC sequence of *FORC*^A ^and not sequences flanking this invariant core are critical for this interaction.

### *FORC*^A ^is a light-responsive promoter element whose activity is modulated by defense-related stimuli

**Figure 2:**
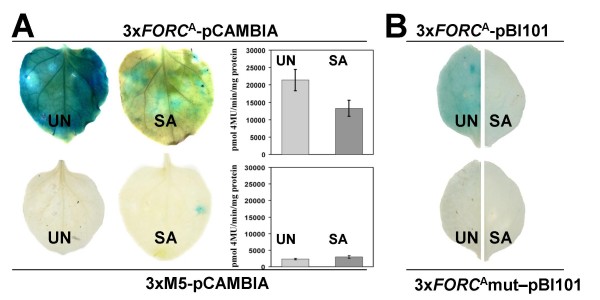
**Agrobacterium-mediated transient expression assays in *N. benthamiana *leaves**. A: GUS histochemical staining and specific activity in *N. benthamiana *leaves after a transient expression assay without (UN) or with (SA) a 24 h salicylic acid treatment using 3x*FORC*^A^-pCAMBIA or 3xM5-pCAMBIA. Mean and standard error of the quantitative data were calculated from at least six individual measurements. Based on T-tests results for 3x*FORC*^A^-pCAMBIA were significantly different (*p *= 0.026), while those for 3xM5-pCAMBIA were not (*p *= 0.42). B: GUS staining in *N. benthamiana *leaves after a transient expression assay without (UN) or with (SA) a 24 h salicylic acid treatment using 3x*FORC*^A^-pBi101 or 3x*FORC*^A^mut-pBi101.

To test effects of *FORC*^A ^on transcriptional regulation, we fused a *FORC*^A ^trimer consisting of three distinct *FORC*^A ^permutations to the minimal *CaMV35S *promoter followed by *GUS *in pCambia1281X (3x*FORC*^A^-pCAMBIA). Untreated *Nicotiana benthamiana *leaves transiently transformed with 3x*FORC*^A^-pCAMBIA by *Agrobacterium tumefaciens *exhibited strong GUS activity after histochemical staining or quantitative GUS activity assays (Figure 2A). Treatment of transformed *N. benthamiana *leaves with 1 mM SA clearly reduced this activity. With a construct containing a trimer of a motif unrelated to *FORC*^A^(3xM5-pCAMBIA) we observed no significant reporter gene activity, while a trimer of a second unrelated motif, M6, mediated a strong increase of GUS activity upon SA treatment (see Additional File 2). Formally the SA-triggered reduction of 3x*FORC*^A^-pCAMBIA expression in our agro-transient assays may be due to effects of SA on the viability of Agrobacterium or other effects unrelated to *FORC*^A^-mediated gene expression. However, we believe that this is quite unlikely for the following reasons. (1) we also observed a SA-triggered reduction of 3x*FORC*^A^-pCAMBIA expression in stably transformed Arabidopsis lines (see below); (2) motif M6 mediated SA-inducibility of *GUS *expression in our agro-transient assays; (3) treatment with 1 mM SA did not reduce *GUS *expression mediated by the constitutive *CaMV35S *promoter in previously published agro-transient assays with tobacco leaves [27,28].

pCAMBIA1281X contains a copy of the full *CaMV35S *promoter close to its multiple cloning site. In order to rule out that this promoter influences the expression pattern observed with 3x*FORC*^A^-pCAMBIA, we constructed a second pair of reporter gene vectors using pBI101 (Clontech), which lacks any functional promoters in the vicinity of its multiple cloning site. The construct 3x*FORC*^A^-pBI101 contains a *FORC*^A ^trimer fused to the minimal *CaMV35S *promoter followed by *GUS*. 3x*FORC*^A^-mut-pBI101 is identical to 3x*FORC*^A^-pBI101, but contains block mutations eliminating the TGGGC core motif of each *FORC*^A ^copy. Consistent with the results observed with the pCAMBIA versions, 3x*FORC*^A^-pBI101 exhibited in agro-transient expression assays a strong constitutive activity that is suppressed by SA, while 3x*FORC*^A^-mut-pBI101 did not show any detectable reporter gene activity (Figure 2B).

**Figure 3:**
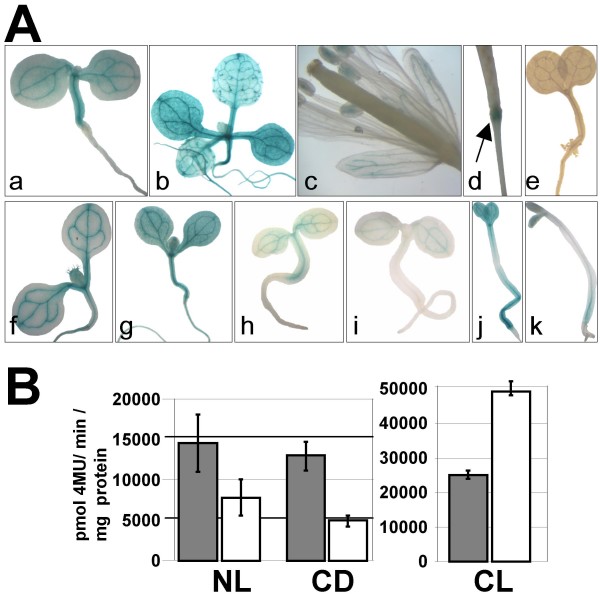
**Reporter gene assays in stably transformed Arabidopsis plants**. A: Histochemical localization of GUS expression in transgenic Arabidopsis plant carrying 3x*FORC*^A^-pCAMBIA. a, 4 day-old seedling; b, 10 day-old plant; c, 30 day-old plant showing GUS staining in pollen grains and sepals; d, Silique showing GUS activity at the top of developing peduncle; e, control 4 day-old seedling carrying the 35Smin-pCAMBIA;f-k, 7 day-old seedlings treated with 0.05% EtOH (f, h and j) or a 24 h SA (1 mM) treatment (g, i and k) under different light conditions: continuous light (f-g), normal light (h-i), or continuous dark (j-k). B: Fluorometric analysis of GUS specific activities in protein extracts of 3x*FORC*^A^-pCAMBIA plants. Ten day-old plants were treated by either continuous light (CL), normal light (NL) or continuous dark (CD) for three days. After two days light treatment, plants were sprayed with 0.05% EtOH (grey bars) or 1 mM SA (white bars) and shock frozen for protein extraction. Mean and standard error were calculated from six pooled data points generated in three independent experiments, each with two transgenic lines. Based on T-tests all differences between mock and SA-treated samples were significant (*p *= 9.82E-06 for CL; *p *= 0.038 for NL and *p *= 0.0053 for CD). Differences between mock-treated CL and NL samples were also significant (*p *= 0.0014).

We also generated transgenic Arabidopsis lines transformed with 3x*FORC*^A^-pCAMBIA. T2 progeny from two independent primary transformants were selected for the presence of the transgene and examined for *GUS *expression by histochemical staining with X-Gluc (Figure 3A). Both tested lines exhibited identical GUS expression patterns. Four day-old 3x*FORC*^A^-pCAMBIA seedlings showed constitutive GUS activity in stems which is most pronounced in vascular tissues (Figure 3A–a). Ten day-old seedlings exhibited 3x*FORC*^A^::*GUS *expression in all plant tissues. In these plants, GUS staining is in particular strongly detectable in vascular tissues and the epidermal tissues surrounding the bases of trichomes (Figure 3A–b). In mature flowers, GUS staining appears in the vascular tissues of sepals and in the anthers, but not in other flower organs (Figure 3A–c). After fertilization, GUS staining is detected at the top of the flower peduncle (Figure 3A–d). No GUS staining was detectable in any of three tested independent T2 lines containing only the *CaMV35 *minimal promoter fused to *GUS *(35Smin-pCambia) (Figure 3A–e). Consistent with results from our EMSAs (Figure 1), seven day-old 3x*FORC*^A^-pCAMBIA plants exhibited stronger GUS accumulation in seedlings grown under CL conditions, compared to either NL- or CD-treated seedlings (Figure 3A–f, 3A–h and 3A–j). Application of SA to seedlings grown under NL or CD regime resulted in clearly reduced reporter gene expression compared to the respective mock-treated controls, (compare Figures 3A–h,  3A–I, 3A–j &3A–k), while under CL conditions SA treatment resulted in enhanced GUS activity (Figures 3A–f &3A–g). This effect was particularly clear in tissues outside the vasculature.

**Figure 4:**
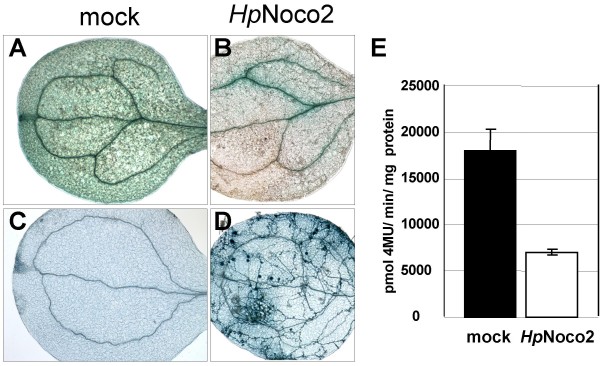
**Infections with virulent *Hyaloperonospora parasitica *suppress *FORC*^A^-mediated transcriptional activity**. A-D: GUS staining (A&B) of plants treated with H_2_0 (A) or *HpNoco2 *(B). Trypan blue staining (C&D) of plants treated with H_2_0 (C) or *Hp*Noco2 (D). E: Fluorometric analysis of GUS specific activity in protein extracts of 3x*FORC*^A^-pCAMBIA plants subjected to mock-treatment or sprayed with *Hp*Noco2 (2*10^4 ^spores/ml). Mean and standard error were calculated from four pooled data points generated in two independent experiments, each with two transgenic lines. Based on T-tests the differences were significant (*p *= 0.0036).

Quantitative assays confirmed that in 3x*FORC*^A^-pCAMBIA plants GUS expression is down-regulated by SA under normal light conditions as well as after 3 days exposure to constant darkness (Figure 3B). However, after constant light treatment, SA triggered a strong increase of reporter gene expression in these lines. Consistent with the SA-induced suppression of *FORC*^A^-mediated GUS expression observed in 3x*FORC*^A^-pCAMBIA Arabidopsis lines under normal light conditions, GUS expression in these lines grown under normal light conditions is also down-regulated after infection with the virulent *Hp *isolate Noco2 (Figures 4A, B &4E). In Col-0 plants, *Hp*Noco2 is known to induce a basal defense response that is dependent on SA [29].

In our assays the high stability of the GUS enzyme in plant tissue [30] resulted already in strong X-Gluc-staining of 3x*FORC*^A^-pCAMBIA seedlings prior to infection with *Hp*Noco2 (not shown). To be able to detect a reduction of this background activity, we had to incubate the seedlings for at least 7 days after spray-infection with *Hp*Noco2 spores. At this timepoint a dense network of *Hp *hyphae had typically developed in cotyledon and leaf tissues. To eliminate the possibility that the reduction of GUS activity in infected tissues is due to disease associated necrosis, we stained *Hp*Noco2-sprayed seedlings 7 dpi with trypan blue, which marks dead plant cells dark blue. As shown in Figure 4D dead plant cells are absent in *Hp*Noco2 infected 3x*FORC*^A^-pCAMBIA seedlings 7 dpi and only trypan blue-stained *Hp *structures are visible in these samples. This indicates that the observed reduction of *GUS *expression is a result of down-regulated 3x*FORC*^A ^activity.

Taken together our reporter gene assays using stably transformed Arabidopsis lines, transiently transformed *N. benthamiana *leaves and EMSAs with nuclear Arabidopsis proteins showed that *FORC*^A ^is a constitutive promoter element, the activity of which is modulated by defense- and light-associated signaling processes.

### Exposure to constant light enhances basal defense to *Hp*

**Figure 5:**
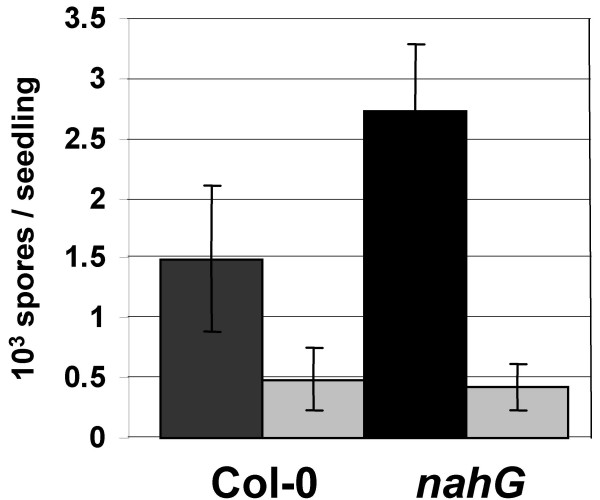
**Continuous exposure to light results in reduced susceptibility to virulent *Hyaloperonospora parasitica***. *HpNoco2 *growth has been evaluated on plants pretreated for 3 days by normal light (dark grey) or continuous light (light grey) exposure. Two weeks old plants were sprayed with *HpNoco2 *(2*10^4 ^spores/ml) and spores were counted 7 dpi. Bars represent mean value of spores/seedling from four different experiments. Based on T-tests differences between normal light and constant light treated samples were significant only for *nahG *plants (*p *= 0.0084).

The existence of crosstalk between defense and light signaling suggested that continuous exposure of Arabidopsis seedlings to light affects the efficiency of their defense responses. Indeed, we observed that Col-0 seedlings, when subject to three days of constant light exposure, appear to exhibit reduced susceptibility to *Hp*Noco2 (Figure 5). This effect is more pronounced and highly significant in *nahG *seedlings, which are compromised in defense-associated SA accumulation and, hence, are hyper-susceptible to *Hp*Noco2 [31]. In both Col-0 and *nahG *seedlings constant light pre-treatment resulted in the same level of protection to *Hp*Noco2, indicating that this effect does not require SA.

## Discussion

Both PTI and ETI are associated with massive transcriptional reprogramming [19,32]. Although multiple types of *cis*-elements and transcription factors controlling defense-related genes have been identified, the important regulatory step of activation of the plant defense transcriptome is still poorly understood. Our current knowledge about defense-associated transcriptional regulation has mainly resulted from the analysis of individual "model genes" such as the Arabidopsis *PR1 *and Parsley *PR10 *genes [33,34]. The vast amount of global transcript profiling data that has accumulated for Arabidopsis and other model plants now allows us to address the identification of such regulatory mechanisms from a new perspective enabling researchers to directly pursue the discovery of transcriptome-level control elements. Multiple studies reported the existence of statistically conserved promoter motifs in clusters of co-expressed genes [20,21,35], but only a few reports provided evidence for their roles as regulatory promoter elements as well as binding sites of nuclear proteins [22,23].

Here we provide another successful demonstration supporting the feasibility of this approach. *FORC*^A^-related motifs have not been implicated in the control of defense-associated gene expression before. We found *FORC*^A ^to DNA-sequence specifically interact with nuclear Arabidopsis proteins. These interactions are differential and suppressed by defense-related stimuli. Furthermore, our data showed *FORC*^A ^to function as a constitutive promoter element, whose activity can be modulated by the defense hormone SA or recognition of *Hp*.

The *FORC *cluster constitutes a set of genes that are coordinately up-regulated in response to infections by oomycete and fungal pathogens. In addition, 19 of the 43 *FORC *genes represented on the Affymetrix ATH1 array are significantly up-regulated by the SA analog BTH [36]. As in Arabidopsis plants grown under a normal light regime the constitutive *FORC*^A ^activity is suppressed by defense-stimuli, including SA, this element cannot be sufficient to control the defense-associated expression of *FORC *genes. The *FORC*-type expression pattern can only be the result of the combined activity of *FORC*^A ^and additional *cis*-elements that act as positive pathogen response elements. We previously reported the enrichment of a W box-like motif and an unknown motif in the promoters of *FORC *genes, which may mediate the pathogen-induced up-regulation of this cluster [15]. Indeed, we found these motifs to exhibit *Hp*-inducible band shifts in EMSAs with nuclear Arabidopsis proteins (not shown), suggesting a positive contribution to transcriptional activity. The role of *FORC*^A ^may be to limit the amplitude of *FORC *gene induction and/or to mediate the down-regulation of *FORCs *after an early peak of their transcript levels is reached. Future experiments will have to address this possibly interesting interplay between different pathogen-response elements.

Both defense-related and light-related stimuli affect the transcriptional output mediated by *FORC*^A^. Under normal light conditions or constant darkness *FORC*^A ^exhibits a constitutive activity that is suppressed by defense-related stimuli. Under constant light-exposure *FORC*^A ^acts as a positive defense-related element mediating SA-inducible gene expression. The latter feature of *FORC*^A^, however, is not reflected in our EMSAs with nuclear Arabidopsis proteins. Under constant light conditions no further increase of the intensity of *FORC*^A ^band shifts was observed after SA treatment (not shown). This may indicate that under constant light conditions *FORC*^A^-dependent pathogen inducibility is not mediated by enhanced binding of nuclear factors, but rather other mechanisms that cannot be detected under our EMSA conditions, such as subtle post-translational modifications of pre-bound factors or transient interactions with co-factors.

**Figure 6:**
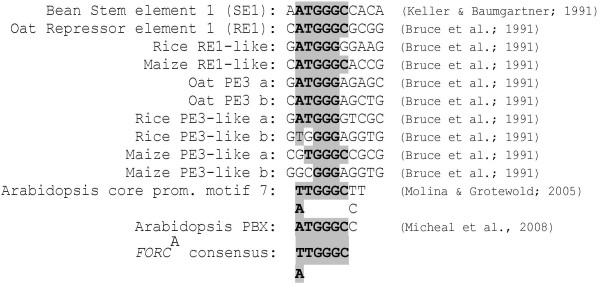
***FORC*^A ^is related to multiple described promoter elements**. Known or hypothetical promoter elements with sequence similarities to *FORC*^A ^are aligned. Positions matching the *FORC*^A ^consensus motif are highlighted in grey.

To our knowledge *FORC*^A ^is unrelated to any described defense-associated promoter elements. Using plant *cis*-element databases (Plant CARE and PLACE, ; ), however, we found several known *FORC*^A^-like promoter motifs that have been implicated in other biological processes (Figure 6). The bean stem element 1 (SE1), that appears to act as a strong unspecific enhancer, and the oat light-repressor element 1 (RE1), which suppresses light-driven reporter gene expression, contain sequences that perfectly match the *FORC*^A ^consensus [37,38]. The positive light response element PE1 from oat contains a sequence that matches 5 of the 6 conserved positions of *FORC*^A ^[38]. RE1 and PE1 are conserved in the promoters of multiple *phyA *genes that are regulated in a light-dependent manner. Inspecting a data set on light triggered transcriptome changes in Arabidopsis [39] we also found *FORC*^A ^to be conserved in the promoters of phytochrome A&B-dependently light-induced genes encoding proteins related to photosynthesis or localized to the chloroplast. In these 26 promoters the *FORC*^A ^hexamer is significantly enriched compared to its statistically expected frequency (*p *= 5.82 E-9). If *FORC*^A ^contributes to the co-regulation of these genes remains to be shown. In addition, a motif that perfectly matches the *FORC*^A ^consensus was found to be conserved in Arabidopsis core promoters [40]. A function has not been assigned to this motif, but its preferential location at the distal periphery of core promoter regions may suggest a role distinct from that of conventional core promoter elements. *FORC*^A ^does also perfectly match the recently identified "protein box" (PBX) which confers phase-specific diurnal and circadian reporter gene expression in Arabidopsis [41]. Promoters that contain PBX are enriched for gene ontology annotations related to protein synthesis.

Taken together, these reports suggest that *FORC*^A^/PBX-related *cis*-elements are conserved throughout the plant kingdom and are generally involved in adjusting the transcriptome to daily changes in light-related (and possibly other) environmental conditions. Furthermore, these *cis*-elements appear to participate in the regulation of a diverse set of genes involved in crucial metabolic processes such as photosynthesis and protein biosynthesis, but also defense. Consistent with such a broad role we observed a 2.9-fold higher frequency of the *FORC*^A ^hexamer in Arabidopsis intergenic sequences than statistically expected. On average, *FORC*^A ^is present 0.78 times per 1 kb in all 1 kb sequences upstream from the 33,282 loci of the TAIR8 Arabidopsis genome annotation , while only 0.27 occurrences of this hexamer per 1 kb are statistically expected in Arabidopsis intergenic regions.

It is presently unclear, what biological purpose it serves that defense-related stimuli reduce *FORC*^A ^activity under normal light conditions or constant darkness, but enhance its activity under constant light exposure. This seemingly unorthodox and contradictory feature of *FORC*^A ^may reflect that in natural environments the probability of plant/pathogen encounters can follow complex diurnal patterns which are further influenced by transient environmental factors, such as local light conditions, temperature, humidity and the presence of leaf surface water [42]. Hence, *FORC*^A ^may act in concert with other regulatory elements to generate fine-tuned gene expression outputs appropriate for a changing array of environmental conditions.

Crosstalk between light and defense signaling has been described in several earlier reports and high light fluence rates or long durations of light exposure have been positively correlated with plant defense reactions [12,13]. For example, Genoud et al. reported interactions between phytochrome-mediated light responses and SA-triggered responses in Arabidopsis [12]. SA-dependent responses such as expression of the defense marker *PR1 *and resistance to avirulent *Pseudomonas syringae *bacteria were found to be triggered by exposure to high fluence rates of white light and to require a functional phytochrome signaling apparatus. It is unclear, however, if the functional characteristics of *FORC*^A ^that we observed are related to the effects described by Gernoud et al., as different light-related parameters were tested in each study. While Gernound et al. varied the fluence rate of white light, we varied the length of the photoperiod. Multiple lesion mimic mutants also point to interactions between light and defense signaling, as they are known to exhibit spontaneous defense responses, such as elevated *PR *gene expression, upon exposure to high light [43,44]. As a possible connection between light and defense signaling in these mutants, light-driven accumulation of reactive oxygen intermediates (ROI), such as superoxide and hydrogenperoxide has been discussed [42,45]. ROI act as an early defense signal and participate in feedback mechanisms modulating SA-dependent defense responses [46,47].

Future experiments will have to address what types of light- and defense signals are important for the crosstalk perceived by *FORC*^A ^and if *FORC*^A^-mediated responses are responsible for the reduced susceptibility to *Hp*Noco2 we observed in Arabidopsis seedlings subject to constant light exposure. A number of *FORC *members appear to show enhanced transcript levels after exposure to a prolonged photoperiod or high light intensity (see Additional File 3). Although this would be consistent with a role of *FORC*^A ^in light-triggered defense responses, further experimentation will be needed to establish such a functional connection. Moreover, it remains to be examined, if the interference between defense and light signaling we have observed is manifested in signal convergence directly at *FORC*^A ^and its cognate transcription factors or in regulatory steps operating further upstream. Alternatively, such crosstalk may not be mediated by discrete signaling steps at all and may be merely a consequence of general disturbances of a highly complex plant signaling network [48]. At this point our knowledge about the architecture and dynamics of regulatory networks in plants is only rudimentary. Emerging new methodologies of computational biology, functional genomics and systems biology will allow us to shift the focus from understanding individual signaling circuits to a more holistic view of regulatory processes. Having promoter elements at hand that integrate signals from multiple input channels will aid in the dissection of such complex regulatory interactions.

## Conclusion

*FORC*^A ^(T/A T G G G C) is a hexameric promoter motif that is conserved in clusters of Arabidopsis genes co-expressed in response to fungal or oomycete pathogens as well as defined light treatments. It constitutively interacts in a DNA-sequence specific manner with nuclear Arabidopsis proteins. These interactions are suppressed by defense-related stimuli and enhanced by prolonged exposure to constant light. Furthermore *FORC*^A ^mediates constitutive reporter gene expression in transiently transformed *N. benthamiana *leaves as well as in stably transformed Arabidopsis plants. Its responsiveness to defense-stimuli is modulated by the duration of light exposure. We propose that *FORC*^A ^integrates light and defense related signals and participates in adjusting the transcriptome to daily changes in light-related environmental conditions.

## Methods

### Plants material and treatments

Arabidopsis ecotype Col-0, transgenic Col-0 *nahG *plants [31] and *Nicotiana benthamiana *plants were grown on soil under fluorescent lights (21°C, 100 E m^-2 ^sec^-1^). Normal light cycles were 14 h day/10 h night. For some experiments, seedlings grown under normal conditions were exposed for additional three days to continuous light or dark treatment. *Hyaloperonospora parasitica *(*Hp*) was grown, propagated and applied to Arabidopsis as previously described [49]. Two week-old seedlings were spray-inoculated with *Hp *spore suspensions (6 × 10^4 ^to 1 × 10^5 ^spores ml^-1 ^of water for *Hp*Hiks1 and *Hp*Emoy2 and 2 × 10^4 ^spores ml^-1 ^for *Hp*Noco2) with Preval paint sprayers. *Hp*Hiks1, *Hp*Emoy2 and *Hp*Noco2 growth was determined 7 dpi by trypan blue tissue staining or visual sporangiophore counts [49]. SA was applied to Arabidopsis and *N. benthamiana *at a concentration of 1 mM in 0.05% EtOH using Preval sprayers.

### Preparation of nuclear proteins and electrophoretic mobility shift assays

Nuclear protein extractions were performed as previously described [50] from 15 g of Arabidopsis whole seedlings that were untreated or pre-treated as described in the legend of Figure 1. Bio-Rad Bradford assays were used to determine protein concentrations. EMSAs were performed using the synthetic oligonucleotides (invitrogen) listed in Figure 1C. Fifty picomoles of double-stranded oligonucleotides were radio-labeled using T4 polynucleotide kinase (NEB) and γ^32^P-ATP. 2.5 picomole G50-purified radioactively labeled double stranded probe (10000 to 30000 cpm) was incubated with 15 μg of nuclear protein in a total volume of 30 μl of binding buffer (20 mM Hepes-KOH pH 7.9; 1.5 mM MgCl_2; _0.2 mM EDTA; 200 mM NaCl; 1 mM DTT) for 20 min at room temperature. For competition experiments, 100-fold molar excess of unlabeled competitor was added to the binding reaction. The EMSA reactions were subjected to electrophoresis on 5.4% non-denaturing polyacrylamide gels in 0.5 × tris borate EDTA buffer (TBE) at room temperature. The gels were vacuum dried and autoradiographed for 5 days at -80°C on HyBlot CL autoradiography film (Denville Scientific).

### Molecular cloning

Trimers consisting of three different *FORC*^A ^permutations (5'-TACGCCGAATTCAAGCTTCTTGTTGGGCCTTAAAAACAATTGGGCAATCAAAAACATTGGGCAGTGTCTAGACTCGAGGATTACGCC-3') or three different permutations of the M5 motif (5'-TACGCCGAATTCAAGCTTCTTTTGAC CATTCTTTTGACCATTCTTTTGACCATTCTCGAGGTCGACGATTACGCC-3') as well as the M6 motif (5'TTGATATCGAATTCCTAAGTGAAGAAGAAACGAGC ATCTTAAGAAGAAGTCGTGACAAAAAAGAAGAAGATCAGGTCTCGAGGTCGACGG-3') were cloned as HindIII-SalI inserts into pBT10 [51]. The resulting 3x*FORC*^A^-pBT10 and 3xM5-pBT10, which include the -46 bp *CaMV35S *minimal promoter (35Smin) present in pBT10 in addition to the respective trimeric promoter motif, were digested with NcoI and HindIII. These HindIII-NcoI inserts were fused to the *GUS *reporter gene in plasmid pCambia1281X to give 3x*FORC*^A^-pCAMBIA, 3xM5-pCAMBIA and 3xM6-pCAMBIA. 3x*FORC*^A^-pCAMBIA was digested with HindIII and SalI. After purification the linearized 3x*FORC*^A^-pCAMBIA was blunt-ended and ligated to give 35Smin-pCambia as a negative control plasmid. 3x*FORC*^A^-pCAMBIA was PCR amplified with pBI-HindIII-F (5'-GATTACGCCAAGCTTGAATTC-3') and pBI-Xba-R (5'-GAAATTTACCTCTAGATCTACC-3'). The PCR product was digested by HindIII and XbaI and introduced into pBi101.1 (Clontech) to give 3x*FORC*^A^-pBi101. 3x*FORC*^A^-pBi101 was digested with HindIII and SalI to remove the 3x*FORC*^A ^sequence. The trimer of *FORC*^A^-mut(5'TACGCCGAATTAAGCTTCCTTGGAATT CCTTAAAAACAAGAATTCAATCAAAAACAGAATTCAGTGTCTAGAGTCGACGATTACGCC-3') was digested with HindIII and SalI, and introduced into 3x*FORC*^A^- pBi101 which was predigested by HindIII and SalI to give 3x*FORC*^A^-mut-pBi101. All restriction digestion products used for cloning (Trimers, PCR products and plasmids) were purified with the QIAquick PCR purification kit (Qiagen). All plasmids have been propagated using *Escherichia coli *DH5alpha. The sequences of inserts and fusion sites with 35Smin and the *GUS *reporter gene in pCambia1281X and pBi101.1 vectors were verified by sequencing for 3x*FORC*^A^- pBT10, 3xM5-pBT10, 3x*FORC*^A^-pCAMBIA, 3xM5-pCAMBIA, 3x*FORC*^A^-pBi101 and 3x*FORC*^A^-mut-pBi101.

### *N. benthamiana *transient expression assays

Transient expression of 3x*FORC*^A^-pCAMBIA, 3xM5-pCAMBIA in *Nicotiana benthamiana *was based on the protocol established by Popescu et al. [52]. Plasmids 3x*FORC*^A^-pCAMBIA, 3xM5-pCAMBIA, 3xM6-pCAMBIA, 3x*FORC*^A^-pBi101, 3x*FORC*^A^-mut-pBi101 and p19 (a viral RNA silencing suppressor from the *tomato bushy stunt virus*) were first transformed into *A. tumefaciens *GV3101 by electroporation. One transformed colony (verified by PCR) for each plasmid was cultured at 28°C until the *A*_600 _reached 1 to 1.5. The bacteria were centrifuged for 15 min at 2,000 *g*, resuspended in infiltration media (10 mM MES, 10 mM MgCl_2_, 200 μM acetosyringone) to a final *A*_600 _of 1.5, and incubated for 3 to 4 h at room temperature. Bacteria were then washed with 1 volume of fresh infiltration media, and resuspended as described above. A mix of a 1:1 ratio (final OD of 0.75) between *Agrobacteria *containing 3x*FORC*^A^-pCAMBIA, 3xM5-pCAMBIA, 3xM6-pCAMBIA, 3x*FORC*^A^-pBi101, 3x*FORC*^A^-mut-pBi101 and those containing p19 were used for infiltration. The infiltration of bacterial mixtures was performed with a 1-ml syringe on the abaxial side of leaves of 3-week-old *N. benthamiana *plants. After 48 h, mock (EtOH 0.05%) or SA treatments were applied and leaves either shock frozen in liquid nitrogen or x-gluc stained (see below).

### Generation of transgenic Arabidopsis lines

35Smin-pCambia and 3x*FORC*^A^-pCAMBIA was introduced in the *A. tumefaciens *strain AGLO2 by electroporation [53]. Agrobacterium-mediated transformation of Col-0 (T_0_) was performed by the floral-dip method [54]. 35Smin-pCambia and 3x*FORC*^A^-pCAMBIA transgenic plants were selected on 0.5 MS/.0.8% agar media containing 50 mg l^-1 ^hygromycinin in the dark for 1 week and then transferred to soil. T2 lines were used for GUS staining and fluorometric analysis.

### GUS histochemical staining

For *N. benthamiana *transient assays, transformed leaves were infiltrated as described above with a solution containing 1 mg ml^-1 ^5-bromo-4-chloro-3-indoyl-β-d-glucuronide (X-Gluc), 50 mm Na_2_PO_4 _pH 7.2, 0.5 mm K_3_Fe(CN)_6_, 0.5 mm K_4_Fe(CN)_6 _incubated O/N at 37°C and cleared with 70% ethanol (EtOH). For Arabidopsis transgenic plants, whole seedlings were vacuum infiltrated for 15 min, incubated O/N at 37°C and cleared with 70% EtOH.

### Fluorometric analysis of GUS activity

Protein was extracted from 15–20 Arabidopsis seedlings or 5–6 *N. benthamiana *leaves in MUG extraction buffer (50 mm sodium phosphate (pH 7.0), 10 mm EDTA (pH 8.0), 0.1% SDS and 0.1% Triton X-100). Ten microliters of crude extract was added to 290 μl of reaction mix containing 10 mM 4-MUG (Gold Biotechnology, St. Louis, MO) in MUG extraction buffer. Reactions were stopped at 15-min intervals by adding 50 μl of MUG reaction mixture to 200 μl 1 M sodium carbonate. Reactions were performed in 96-well plates at 37°C. Fluorescence was measured at an excitation wavelength of 365 nm and emission wavelength 455 nm using Wallac 1420-012 Multilabel Counter (PerkinElmer Life Science). Protein concentrations in extracts were determined using Bio-Rad protein assays following the manufacturer's instructions (Bio-Rad Laboratories). The GUS activity was expressed as picomoles of 4-methyl umbelliferone per milligram of protein per minute. Final values were adjusted to eliminate background fluorescence by subtracting the average GUS activity measured from non-β-glucuronidase (uidA, GUS) expressing Arabidopsis seedlings. For *N. benthamiana*, GUS activity values are expressed as means of 5–10 samples each containing 5–6 leaves for three experimental repetitions. For GUS expressing transgenic Arabidopsis lines GUS activity values are means of 5–10 samples each containing 15–20 seedlings from two independent lines for three independent experimental repetitions.

### Statistical analyses of conserved promoter motifs

*P*-values describing the enrichment of defined sequence motifs in promoter sets were calculated using the Poisson distribution as described previously [15]. However, due to the general conservation of *FORC*^A ^in a large number of Arabidopsis promoters, the expected frequency of this motif was calculated based on theoretical assumptions and not based on its actual occurrence in all Arabidopsis promoters as previously [15]. Arabidopsis intergenic regions contain approximately 16% "Cs", 16% "Gs", 32%"As" and 32% "Ts". Based on this the expected frequency of the *FORC*^A ^hexamer (A/T T G G G C) approximately equals 0.64 × 0.32 × 0.16^4 ^× 2 × 1000 = 0.27 occurrences on both strands of 1 kb of random intergenic DNA.

## Authors' contributions

AE designed and performed all experiments and wrote parts of the manuscript. NT performed the statistical analyses. TE supervised this project and wrote parts of this manuscript.

## Supplementary Material

Additional file 1 **Arabidopsis *FORC *genes are co-expressed in response to infections with the oomycete *Phytphthora infestans *and the necrotrophic fungus *****Botrytis cinerea***. Represented are transcript levels triggered in Arabidopsis (accession Col-0) infected by *Phytophthora infestans *(*Pi*) or *Botrytis cinerea *(*Bc*). Transcript levels are illustrated as ratios between infected and mock treated control samples (red signal signifies an up-regulation in infected tissue relative to the control). All transcript data were generated using Affymetrix ATH1 whole genome arrays and were downloaded in analyzed form [25] from the Botany Array Resource web site . They were provided by Dierk Scheel, Frederic Brunner & Lore Westphal (*Pi*) as well as Carine Denoux, Fred Ausubel, Julia Dewdney & Simone Ferrari (*Bc*). Click here for file

Additional file 2 **Agrobacterium-mediated transient expression assays with 3xM6-pCAMBIA in *N. benthamiana *leaves**. GUS staining in *N. benthamiana *leaves after a transient expression assay without (UN) or with (SA) a 24 h salicylic acid treatment using 3xM6-pCAMBIA. Shown are typical examples of *N. benthamiana *leaves. Click here for file

Additional file 3 **Transcript levels of Arabidopsis *FORC *genes are affected by photoperiod and light intensity**. Represented are relative transcript levels of *FORC *genes after exposure of Arabidopsis plants to different photoperiods or 3 h-exposure to a high fluence rate of 1000 uM m^-2 ^s^-1 ^white light (in Col-0 wild type, *cry1 *or *hy5 *mutant plants). All transcript data were generated using Affymetrix ATH1 whole genome arrays and were downloaded in analyzed form [55] from the Genevestigator web site . Transcript levels are illustrated as ratios between plants exposed to an 8 h photoperiod and plants exposed to a 16 h photoperiod (Photoperiod 8 h/16 h) or as ratios between plants exposed to high white light fluence rates versus control treated plants (high light). The scale bar on the left hand side relates color intensity to linear fold-change values. For the "photoperiod data set" green signal signifies an up-regulation in plants exposed to the longer photoperiod relative to plants exposed to the shorter photoperiod. For the "high fluence rate data set" red signal signifies an up-regulation triggered by high light intensity. The data were generated by M. Schmid and D. Weigel (photoperiod) and Kleine et al. [56] (high fluence rate). Click here for file
