# Supplementary figures and images for: FORCA, a promoter element that responds to crosstalk between defense and light signaling
Source: BMC Plant Biol. 2009 Jan 7;9:2. doi: 10.1186/1471-2229-9-2 (PMC2640394; doi:10.1186/1471-2229-9-2)

## Slide 1
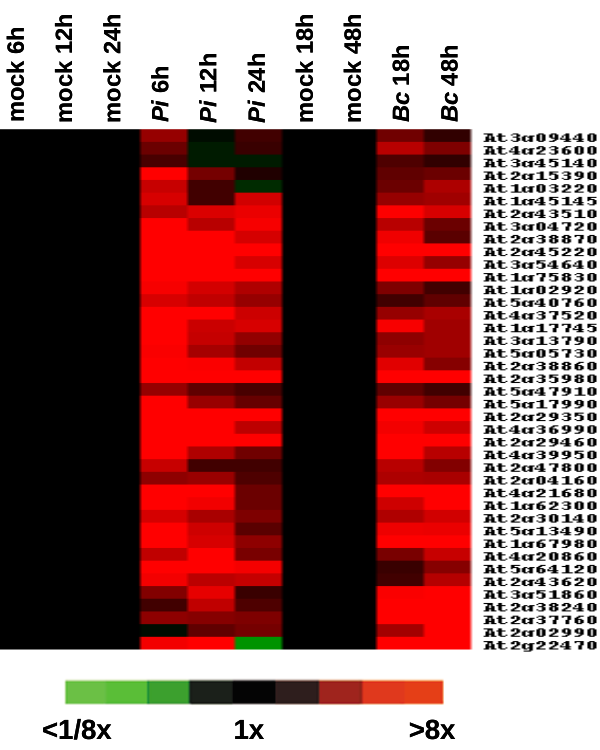

Supplement: Additional file 1 — Arabidopsis FORC genes are co-expressed in response to infections with the oomycete Phytphthora infestans and the necrotrophic fungus Botrytis cinerea. Represented are transcript levels triggered in Arabidopsis (accession Col-0) infected by Phytophthora infestans (Pi) or Botrytis cinerea (Bc). Transcript levels are illustrated as ratios between infected and mock treated control samples (red signal signifies an up-regulation in infected tissue relative to the control). All transcript data were generated using Affymetrix ATH1 whole genome arrays and were downloaded in analyzed form [25] from the Botany Array Resource web site . They were provided by Dierk Scheel, Frederic Brunner & Lore Westphal (Pi) as well as Carine Denoux, Fred Ausubel, Julia Dewdney & Simone Ferrari (Bc). [file 1471-2229-9-2-S1.ppt]

## Slide 1
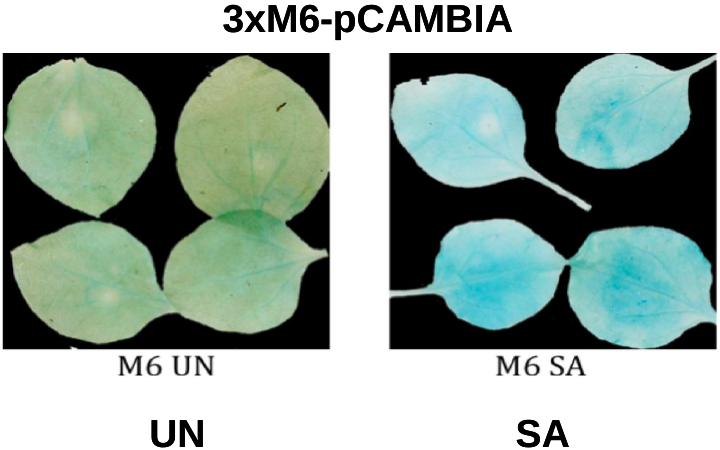

Supplement: Additional file 2 — Agrobacterium-mediated transient expression assays with 3xM6-pCAMBIA in N. benthamiana leaves. GUS staining in N. benthamiana leaves after a transient expression assay without (UN) or with (SA) a 24 h salicylic acid treatment using 3xM6-pCAMBIA. Shown are typical examples of N. benthamiana leaves. [file 1471-2229-9-2-S2.ppt]

## Slide 1
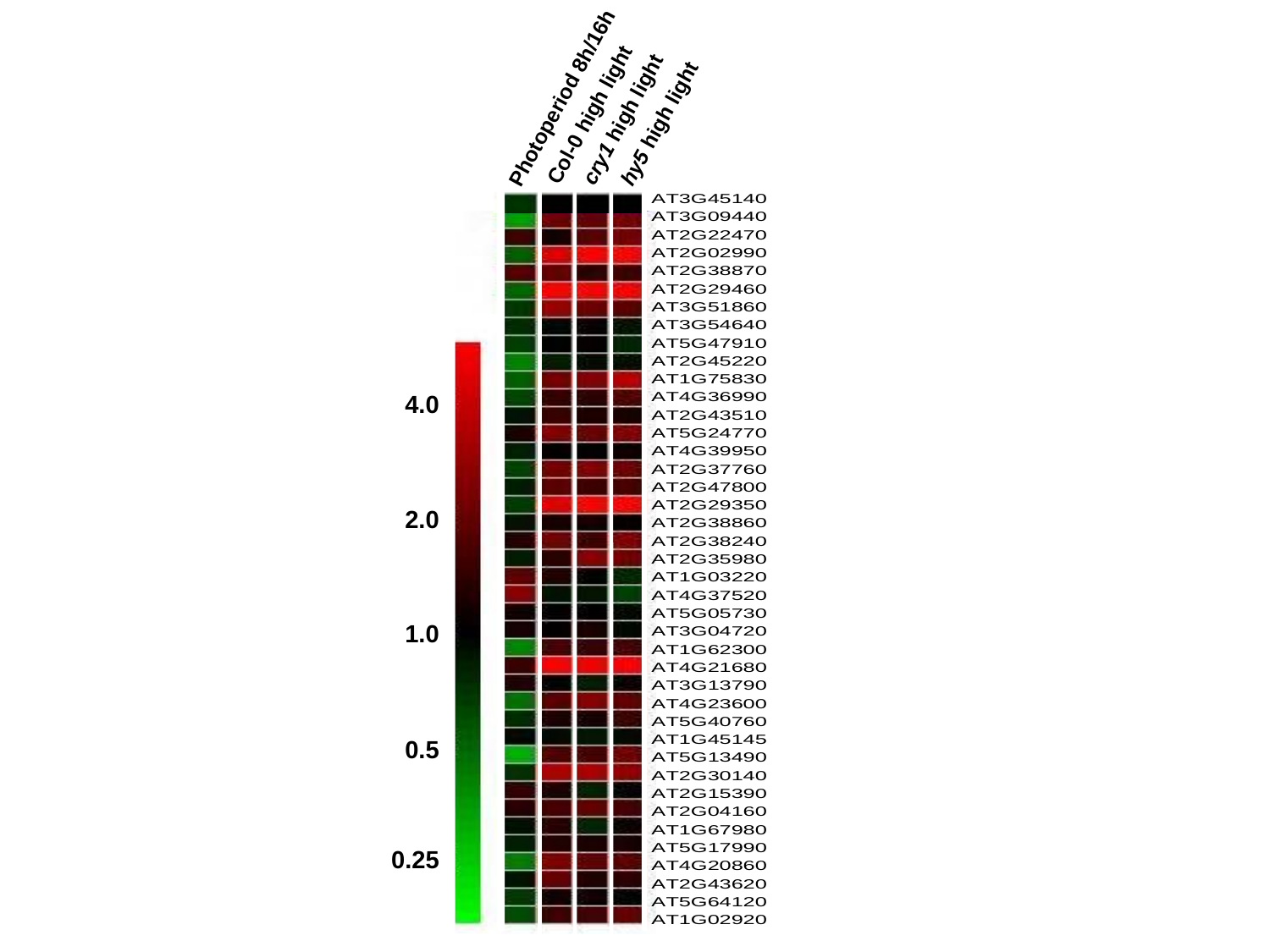

Photoperiod 8h/16h
Col-0 high light
cry1 high light
hy5 high light
4.0
2.0
1.0
0.5
0.25

Supplement: Additional file 3 — Transcript levels of Arabidopsis FORC genes are affected by photoperiod and light intensity. Represented are relative transcript levels of FORC genes after exposure of Arabidopsis plants to different photoperiods or 3 h-exposure to a high fluence rate of 1000 uM m-2 s-1 white light (in Col-0 wild type, cry1 or hy5 mutant plants). All transcript data were generated using Affymetrix ATH1 whole genome arrays and were downloaded in analyzed form [55] from the Genevestigator web site . Transcript levels are illustrated as ratios between plants exposed to an 8 h photoperiod and plants exposed to a 16 h photoperiod (Photoperiod 8 h/16 h) or as ratios between plants exposed to high white light fluence rates versus control treated plants (high light). The scale bar on the left hand side relates color intensity to linear fold-change values. For the "photoperiod data set" green signal signifies an up-regulation in plants exposed to the longer photoperiod relative to plants exposed to the shorter photoperiod. For the "high fluence rate data set" red signal signifies an up-regulation triggered by high light intensity. The data were generated by M. Schmid and D. Weigel (photoperiod) and Kleine et al. [56] (high fluence rate). [file 1471-2229-9-2-S3.ppt]
